# Ectopic pheochromocytomas in the third trimester: A case report and literature review

**DOI:** 10.1097/MD.0000000000036127

**Published:** 2023-02-02

**Authors:** Lei Zhao, Miaomiao Chen, Xiaohong Chen, Ling Yu, Shu Guo Du, Quan Gan, Wen Zhong Yang, Chengcheng Jiang, Mei Xiao

**Affiliations:** a Department of Obstetrics, Maternal and Child Health Hospital of Hubei Province, Tongji Medical College, Huazhong University of Science and Technology, Wuhan, P.R. China; b Department of Internal Medicine, Maternal and Child Health Hospital of Hubei Province, Tongji Medical College, Huazhong University of Science and Technology, Wuhan, P.R. China; c Department of Anesthesiology, Maternal and Child Health Hospital of Hubei Province, Tongji Medical College, Huazhong University of Science and Technology, Wuhan, P.R. China; d Department of Intensive Care Unit, Maternal and Child Health Hospital of Hubei Province, Tongji Medical College, Huazhong University of Science and Technology, Wuhan, P.R. China; e Department of Medical Imaging, Maternal and Child Health Hospital of Hubei Province, Tongji Medical College, Huazhong University of Science and Technology, Wuhan, P.R. China.

**Keywords:** ectopic pheochromocytomas (paraganglioma), perinatal management, perioperative management, pregnancy

## Abstract

**Introduction:**

To investigate the clinical features, pregnancy care, timing, and approaches of pregnancy termination as well as the perinatal management of pregnant women with ectopic pheochromocytomas (EPCC) (paragangliomas, PGL).

**Methods:**

We report the diagnosis and treatment of a pregnant women with EPCC which was confirmed in the third trimester in our hospital. Literature in relation to EPCC during pregnancy both in and outside China was searched for data analysis such as maternal clinical features and maternal and fetal prognosis.

**Results:**

A total of 20 papers including 21 cases (plus ours) were retrieved. The average age of pregnant patients was 28 years old (from 21 to 37). Two patients presented no hypertension. Nineteen had hypertension in various extent with the accompany of headache (11 cases, 57.9%), palpitations (8 cases, 42.1%), sweating (6 cases, 31.6%), nausea (6 cases), abdominal pain (2 cases), etc. The tumor was found in the chest in 3 patients, in the upper abdomen in 1 patient, in the middle abdomen in 10 patients, between the lower abdomen and pelvic cavity in 3 patients and in the pelvic cavity in 3 patients. Five patients had a surgical removal of the tumor before delivery, 3 during cesarean section and 10 after giving birth.

**Conclusion:**

EPCC (PGL) during pregnancy is a rare extra-adrenal tumor, whose manifestations are often confused with those of pregnancy-induced hypertension. It is extremely hard to diagnosis the disease before surgery. Patients still have an opportunity of undergoing spontaneous delivery if their tumors have been removed before labor. However, for patients whose pheochromocytomas is localized before labor, it is better to terminate their pregnancy via cesarean section in a proper time according to their obstetric conditions, while under the supervision of multidisciplinary specialists. The preparations of both α and β adrenergic receptor blocker treatment that is normally carried out before PGL removal surgery are unnecessary to be overemphasized before the cesarean section.

## 1. Introduction

Extra-adrenal pheochromocytomas, also known as paraganglioma (PGL), are rare neuroendocrine tumors derived from chromaffin cells. Most pheochromocytomas (PCC) are extremely rare solid tumors stemming from catecholamine-producing fetal neural crests,^[1]^ with the prevalence of 1 in 100,000 cases.^[2]^ PGL is not often diagnosed or identified during pregnancy and may even leave severe consequences if not treated properly. The clinical manifestations of PGL rely on its location, size and hormone secreting situations. PGL is located very adjacent to the major blood vessels and the peripheral organs, making it more challenging to be diagnosed and treated. Multidisciplinary support for the diagnosis and treatment is necessary. We now report a pregnant woman who was diagnosed with PGL in the third trimester in our hospital and in the meantime review literature on the subject for the purpose of increasing the knowledge and advancing the diagnosis and treatment as well as general management of the disease.

## 2. Clinical data

A 28-year-old pregnant woman who was admitted to our Department of Internal Medicine on November 17, 2021 due to “confirmation of headache at night lasted at least half a month and an elevated blood glucose for over 2 months in our Department of Obstetrics at 34^+1^ weeks of gestation.” The patient was identified with abnormal oral glucose tolerance test (5.16, 12.98, and 12.82 mmol/L) accompanied by occasional thirsty, polydipsia, polyphagia, and polyuria in a prenatal examination in one institution. She only reduced her blood glucose level just by keeping a proper diet and exercising, but failed to control it under limits due to the lack of medication. Half a month later, the patients developed headache at night, which was more obvious when lying down, accompanied by blurred vision, palpitations, and nausea. But in the daytime, she did not experience blurred vision, numbness in extremities, and body itching, hence decided not to seek medication as supposed to. *Checks upon arrival*: T: 36.1 °C, P: 93 beats per min, R: 18 beats per min, and BP:121/ 93 mm Hg; she had cutaneous or sclera icterus, supple neck and regular heart rhythm without sounds of etiological murmurs in any valve; she also had a soft but swollen abdomen free of point and rebound tenderness. The liver and spleen were not palpable under rib cage. The Murphy sign and the bi-renal percussive pain was both negative. No edema was observed in both lower extremities. *Obstetric conditions*: the patient’s fundal height was 28 cm, circumference of abdomen 86 cm, with fetal head half in the pelvic and a fetal heart rate of 121 bpm. *Obstetric ultrasound*: the ultrasound on November 17, 2021 in our hospital revealed a singleton live fetus, head presentation, a biparietal diameter of 8.4 cm, circumference of abdomen of 29.9 cm, amniotic fluid diameter of 4.9 cm, umbilical artery blood flow of 2.30 and an estimated fetal body weight of 2272 g. U-shaped impression was observed in the fetal neck.

**Figure 1.**
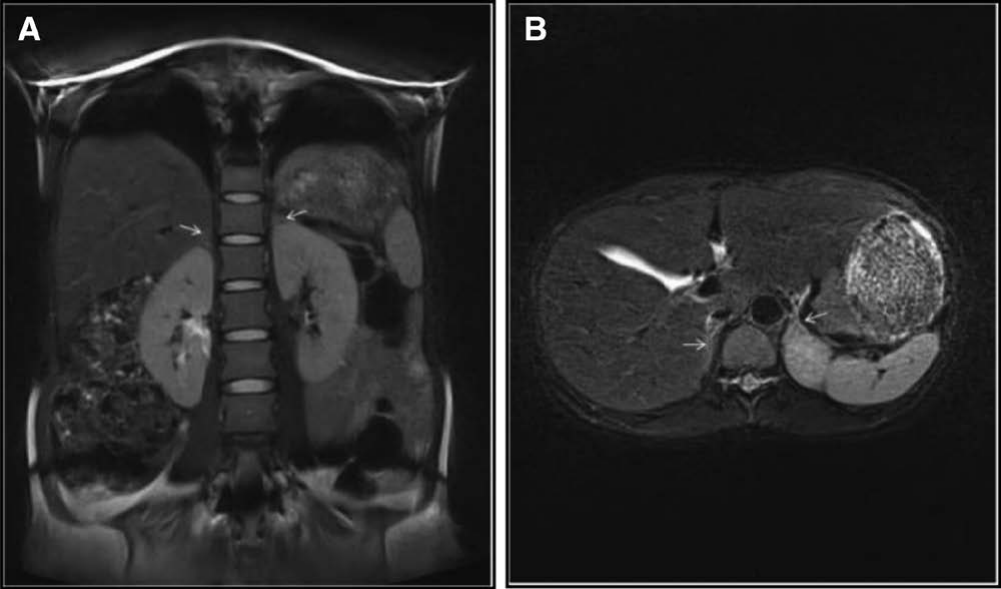
Antenatal MRI scan in the adrenal gland. (A) In the fat-suppressed coronal position image, the junction region between the 2 glands was obvious (as noted by the white arrow), without the presence of abnormal shape or signals. (B) In the fat-suppressed and T1W1 position image, the boundary of the 2 glands was clear, with the lateral limb in the right gland being smooth and linear and that in the left gland being triangular. No abnormal shapes or signals were observed (as noted by the white arrow).

During hospitalization, her blood pressure was monitored, in which paroxysmal fluctuations were found. Headache in the early morning resulted in her systolic blood pressure rising to 206 mm Hg and diastolic blood pressure to 140 mm Hg. The patients denied a history of hypertension. *Other examinations after hospitalization*: the patients had an increased alanine aminotransferase level of 484 U/L, an increased aspartate aminotransferase level of 343 U/L, a reduced albumin level of 35.5 g/L, and an elevated total bile acid level of 16.6 μmol/L; she also had an increased lactate dehydrogenase level of 381 U/L, an elevated α-hydroxybutyrate dehydrogenase level of 278.00 U/L; an increased total cholesterol level of 5.98 mmol/L, an increased low-density lipoprotein cholesterol level of 3.28 mmol/L; she was suggestive of PCC due to the presence of paroxysmal hypertension; her vanillylmandelic acid (VMA) rose to 68.40 mg/24 hours (reference value: 0–12); her glycosylated hemoglobin was 5.90%, 24-hour urine protein increased to 421.37 mg/24 hours; and the 4 results of liver fiber revealed that her hyaIuronicacid level rose to 104.60 ng/mL, iaminin level to 52.4 ng/mL, N-terminate procollagen before type III to 148 ng/mL and collagen type IV to 81.4 ng/mL. On November 21, 2021, the patient underwent ultrasound on liver, gallbladder, spleen, pancreas and kidney as well as MR plain scan in adrenal gland, the results revealed no significant abnormalities (Fig. 1). *Treatments after admission*: due to unsatisfactory blood glucose test result on admission, the patient received subcutaneous injection of 4 U of NovoRapid Flexpen before each meal to control her blood glucose at a standard level. She underwent antihypertensive treatment with β-blocker labetalol (100 mg once every 12 hours at 10 am and 10 pm) and ca^2+^ channel blocker Adalat tablets (30 mg once daily at 6 pm) for 7 days, until her blood pressure was controlled between 134/99 and 153/100 mm Hg. She then received intravenous infusion of Glutathione buccal tablets and Ademetionine 1,4-butanedisulfonate for injection to protect the liver, and Dexamethasone to treat the fetal lung.

**Figure 2.**
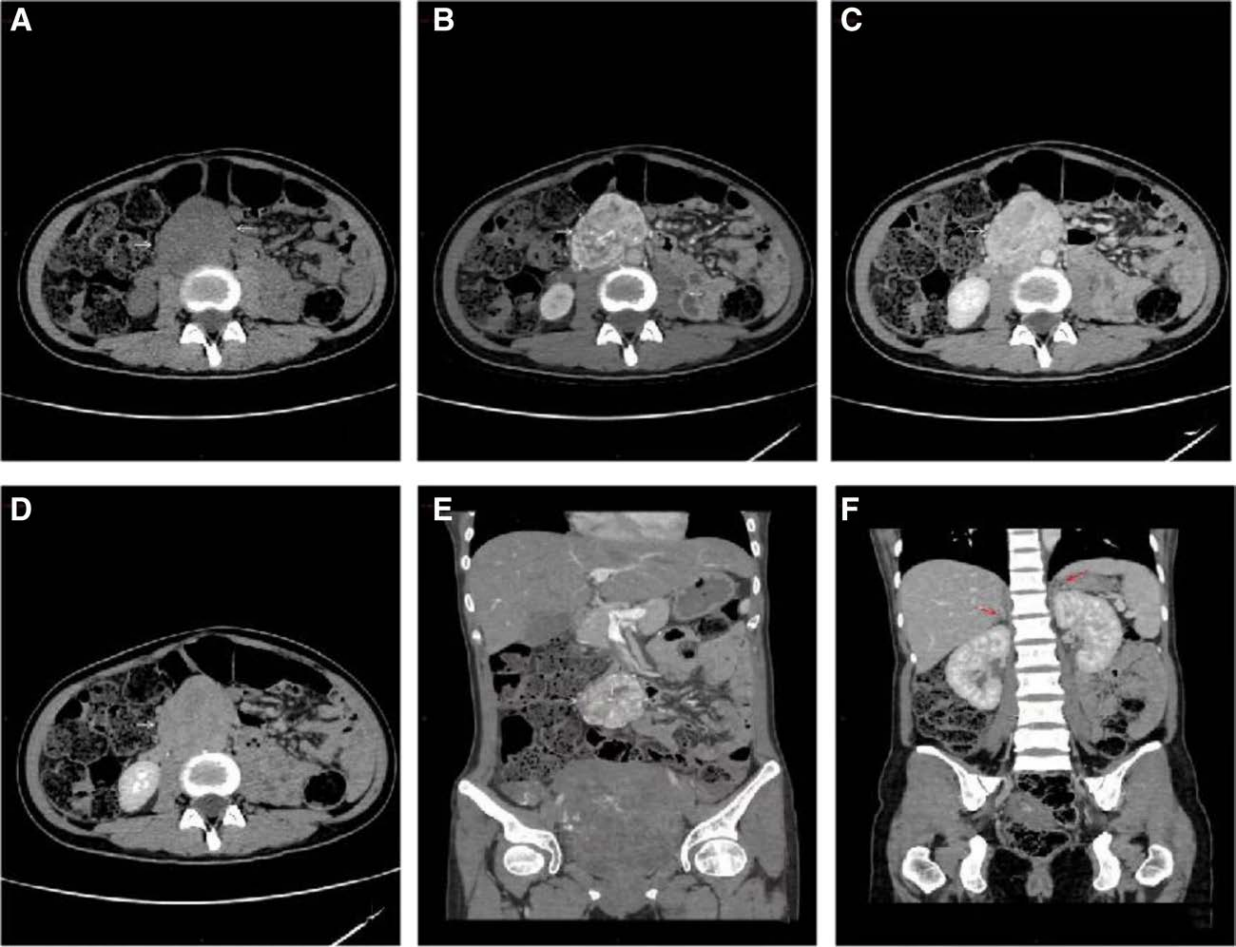
Postpartum CT scan plus enhancement scan in the entire abdomen. (A) In the axial-positioned abdominal CT image, a soft tissue density mass on the right anterior the abdominal aorta (at the very bottom of the left kidney that levels to the L4 spine) was visualized (as noted by the white arrow). The mass, measuring 6.0 cm × 4.5 cm × 6.6 cm, had a clear boundary that gradually turned to a shallow lobe. The CT value was 35HU. (B, C, D, E) In the enhanced three-phase and axial-positioned abdominal CT scan images, and the enhanced portal venous phase and coronal-positioned images, enhancement scan results at the abdominal aorta indicated that irregular changes were markedly observed in the mass, which enhanced significantly in arterial phase but reduced in portal venous phase and equilibrium phase. The CT value was 135HU, 92HU and 60HU in respective. Inside the abdomen seen a weakly enhanced small patches (as noted by the white arrow). (F) In the coronal-positioned enhanced CT scan image of portal venous phase, the shapes and density of the junction line between the 2 glands and their middle and lateral limbs showed no notable abnormality (as noted by the red arrow). CT: computed tomography.

She presented secondary hypertension at 35 weeks of pregnancy on November 24 which was suggestive of PCC. Subsequently, she was transferred to the Department of Obstetrics in the same afternoon and confirmed with PCC through joint-diagnosis by specialists from multidisciplines. However, the tumor was not localized. On November 25, fetal heart rate monitoring showed type II pattern, and then cesarean section was performed under combined spinal-epidural anesthesia due to “secondary hypertension (PCC?), placental dysfunction, liver dysfunction, and intrahepatic cholestasis during pregnancy.” At 09:26 on November 25, 2021, a live male baby was delivered in the left occiput transverse position, with Apgar scores of 9 points after 1 minute, 10 points after 5 minutes, body weight of 2260 g and body length of 47 cm. The entire placenta was spontaneously delivered out from the uterus with level III contamination of amniotic fluid. The intraoperative blood pressure fluctuated between 91 and 216 mm Hg and between 60 and 115 mm Hg. The blood pressure reached the highest to 216/115 mm Hg upon delivery, but lowered under 90/60 mm Hg transiently after intravenous infusion of phentolamine, and restored to normal after labor. The 20-minute surgical process went very well. The anesthesia was ideal during labor, with liquid supplementation of 1000 mL, blood loss of 200 mL and urination of 150 mL in light color. Her postoperative blood pressure was 125/75 mm Hg and pulses were 80 beats per min. She was then sent to Intensive Care Unit for treatment. On November 28, 2021, her reexamination results showed that her VMA level rose to 147 mg/24 hours. Her test results in Picture Archiving and Communication System on November 30, 2021 indicated that:1. No significant abnormality in cranial computed tomography (CT) scan, but additional magnetic resonance imaging (MRI) scan should be performed when necessary; 2. chest CT scan presented no marked abnormality; 3. calcification in the right lobe of liver was observed, suggesting parenchymal calcification or hepatolithiasis. 4. Soft tissue intensity shadow was seen in the slightly right part of the middle abdomen, with clear boundary and a size of 6.0 cm × 4.5 cm × 6.6 cm. Added clinical manifestations the patient presented, she was suspected of ectopic pheochromocytomas (EPCC) (Fig. 2). Her stitches were removed on day 7 after cesarean section and then she was discharged. *Diagnosis on discharge*: patients were confirmed with secondary hypertension (EPCC), placental insufficiency, hepatic insufficiency, bile accumulation in the liver during pregnancy, pregnancy-induced diabetes mellitus, entangled umbilical cord, the delivery of one of the 2 fetuses who was male and live in left occiput transverse position at 35^+1^ weeks of gestation, undergoing selective cesarean section, live singleton, and hypoproteinaemia before discharging from our hospital.

**Figure 3.**
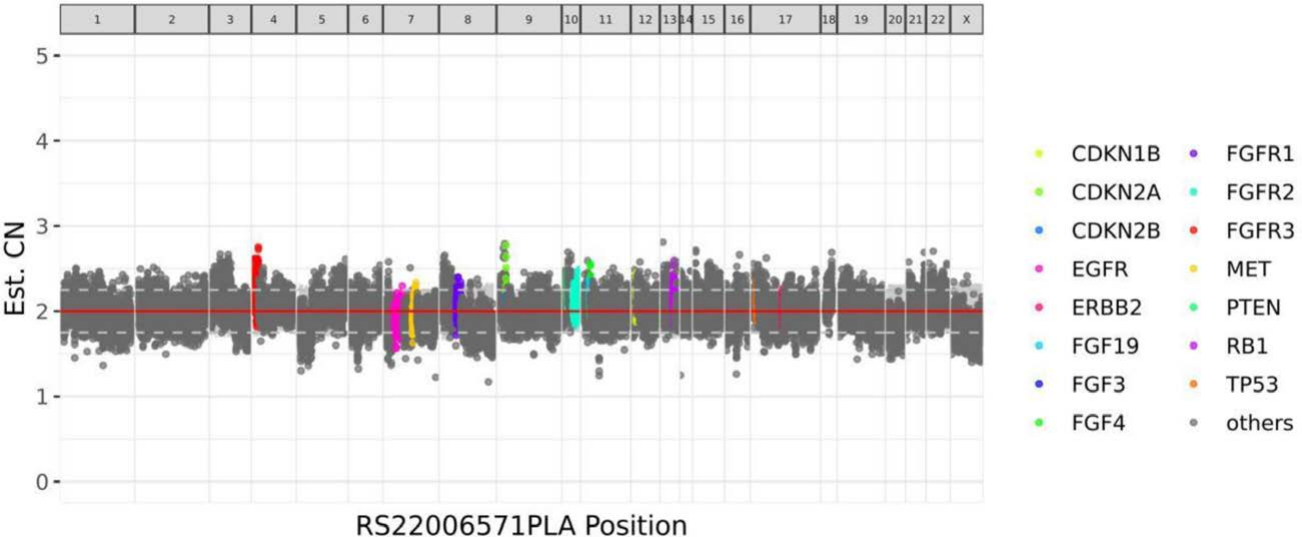
Distribution of genetic copies.

On January 2, 2022, 38 days after labor, the patient was admitted to the Department of Urology, Tongji Hospital, Tongji Medical College of HUST, because of the presence of hypertension for nearly 2 months and EPCC for over a month. On January 4, 2022, enhancement CT scan in the entire abdomen (the upper abdomen, lower abdomen and pelvic cavity) plus Tomography noted a soft tissue mass anterior to the inferior vena cava, suggesting the presence of neoplastic lesions. Her cortisol was 29.6 µg/dL, adrenaline was 9.9 pg/mL, active renin was 40.6 µIU/mL, aldosterone was 91.2 pg/mL, aldosterone to renin ratio was 2.246. *Determination of CA and the metabolites*: plasma norepinephrine rose to 12.90 nmol/L, VMA grew to 127.4 µmol/24 hours. After undergoing treatment with β-adrenergic receptor blocker, the patient was performed with robot-assisted laparoscopic resection of retroperitoneal lesions (EPCC) on January 8, 2022. *Postoperative pathological tests*: (retroperitoneal) extra-adrenal PGL. The results of immunohistochemical tests were CgA (+), Syn (+), CD56 (+), S-100 (Sertoli cells +), SOX10 (Sertoli cells +), GATA-3 (+), SDHB (weak +), PCK (−), Ki-67 (Li about 3%). On January 23,2022, the genetic test report showed that B7-H1 was negative; no class I, II, or III mutations were detected in somatic cells. Figure 3 shows the distribution details of genetic copies.

The patient was followed up for 1 year, during which her blood pressure and blood glucose were both normal, unnecessary of any control measures to keep them at a standard level.

## 3. Literature review

Terms such as extra-adrenal pheochromocytomas, PGL, paraganglioma, extra-adrenal pheochromocytomas and pregnancy in Chinese were searched in Wangfang and CNKI sources, and in English were retrieved from Pubmed database. The publication time of literature was set between January 1996 and December 2021. All papers were gone through to exclude the ones about literature review and repeated cases. In the end, only 20 papers that reported PGL during pregnancy or PGL alone were remained, which included 21 cases confirmed through pathological tests (ours included). See Table S1, Supplemental Digital Content, http://links.lww.com/MD/K755. The following are features of PGL patients during pregnancy summarized from the cases:

1. General data. The average onset age of PGL during pregnancy is 28 years old (between 21 and 37). One cased was diagnosed with PGL during in vitro fertilization oocyte retrieval before conception. The PGL was located in the middle of the ovary (case 2). Four patients were diagnosed in the first trimester and 4 in the second trimester, among which one patient was recurrence. Six patients were confirmed in the third trimester and 5 after delivery.
2. Clinical manifestations. Two patients presented no hypertension whose PGL were both in the middle ovaries. Nineteen patients produced hypertension in various degrees, among which the most common “triad” was headache (11 cases, 57.9%), palpitations (8 cases, 42.1%) and sweating (6 cases, 31.6%). The other symptoms presented included nausea (6 cases), abdominal pain (2 cases), loss of appetite (2 cases), intrahepatic cholestasis of pregnancy cutaneous icterus (2 cases), painless hematuria (1 case) and postpartum high fever (1 case).
3. Complications/comorbidity and other factors. Three patients occurred with pregnancy-induced diabetes, 2 had intrahepatic cholestasis of pregnancy, one produced thrombocytosis and one polycythemia. One patient chose to experience assisted reproductive technology.
4. Preparations before labor. From case 8, 10, and 19 in Table 1, we could learn that it is hard to maintain pregnant if blood pressure is not kept under limits, easily resulting in miscarriage and immature delivery. Seven patients whose tumors were removed during pregnancy had controlled blood pressure and full-term pregnancy. Thirteen patients chose not to remove their tumors while pregnant. Table 1 has listed the antihypertensive drugs these patients used. However, 7 of them who gave birth from 33^+5^ weeks to 40 weeks of gestation did not administer α-blocker to control blood pressure. One patient whose medication was unknown terminated pregnancy through cesarean section at the 28th week of gestation because of above-limit blood pressure. Case 21 was found to have hypertensive crisis while removing ovarian tumor during cesarean section, which was later identified as EPCC through pathological tests. She neither had hypertension nor used α-blockers before surgery. The best gestational week for cesarean section is in relation to the condition of PPGL patients. As the reproductive technology advances, it is beneficial for PPGL patients to choose to end pregnancy at a proper time.
5. Pregnancy outcomes. Four out of the 21 patients had not clear pregnant outcomes. Two underwent cesarean section before the 32th week of pregnancy. Four patients experienced immature delivery after 32 weeks of gestation all via cesarean section. Seven had full-term delivery, among them 3 chose spontaneous labor and were removed of the tumor before delivery. Two patients underwent tumor removal in the second trimester (case 4 and case 7), and one at the 32th week of pregnancy (case 11).
6. The timing and approaches for tumor removal. Once patient was found to have tumor in the ovary while undergoing in vitro fertilization oocyte retrieval, and had it removed during the process. Five patients were diagnosed with PGL in pregnancy, but selected to receive treatment first for controlling the symptoms and remove the tumor at another proper time before delivery. Three patients incised the tumor during cesarean section (case 10, case 15, and case 21). Ten patients experienced surgical tumor removal after labor. One patient had PGL extensively adhered to the aorta and nearby organs, thus chose to receive radioisotope targeted therapy instead of surgical treatment. One patient did not report the time of her tumor removal.
7. Tumor location. Three patients whose PGLs were located in the chest, 2 in the upper abdomen, 6 in the middle abdomen, 6 from the lower abdomen to the pelvis, 4 in the pelvis. Multiple occurrences were found in one patient and recurrence in another patient.

**Table 1:** Antihypertensive medications of pregnant patients who did not undergo EPCC removal before delivery.

NO.	Time from diagnosis to delivery	Antihypertensive drugs				Note (other drugs)
		α-blocker	β-blocker	Ca^2+^ channel blocker	ACEI	
1	6 days		Labetalol	Adalat (nifedipine)		Magnesium sulfate
3^[11]^	33 weeks		Labetalol	Nifedipine (adalat)		Intolerance to prazosin
9^[12]^	14 weeks	Phenoxybenzamine	Propranolol			
10^[13]^	Recurrence during pregnancy	Benzylamine	Propranolol			The therapeutic effects of compound hypotensive tablet, prazosin and magnesium sulfate were not ideal. Tumor removal during cesarean section
12^[14]^	3 weeks	Phenoxybenzamine				
13^[15]^	1 day		Labetalol			Hydralazine and magnesium sulfate
14^[16]^	The very induction day		Labetalol			Hydralazine
15^[17]^	2 weeks	Phenoxybenzamine				Tumor removal during cesarean section
16^[18]^	After delivery	Constant use				Unspecified medication
17^[19]^	After delivery			Nifedipine (adalat)	Enalapril	Hydrochlorothiazide
18^[20]^	After delivery					Magnesium sulfate
19^[21]^	After delivery					Unspecified medication. cesarean section at 28 weeks of gestation due to hypertension
20^[22]^	After delivery	Phenoxybenzamine	Metoprolol			
21^[23]^	Diagnosed through pathological tests after delivery					No hypertension during pregnancy, but occurred during cesarean section and ovarian tumor removal at the same time. No medication before tumor removal

## 4. Discussion

In 1886, Felix Fränkel, a German physician, reported a special clinical and morphological discovery about a mass in the adrenal glands of a 18-year-old girl with PCC for the first time. PCC originates from the adrenal medulla and PGL from the sympathetic nerve chain outside the adrenal glands. Collectively they are called PPGL. PPGLs are neuroendocrine tumors with the ability to secret hormones. It is capable of synthesizing, secreting, and releasing enormous amounts of CA, such as norepinephrine (NE), epinephrine (E), and dopamine (DA). The major clinical manifestations of PPGL are hypertension caused by increased CA and cardiovascular complications and metabolic changes in the heart, brain, and kidney. At present, there is no exact data on the incidence or prevalence of PPGL in China. However, outside China, the data are 2 to 8 patients per million people per year.^[3]^ And the incidence is even lower for patients during pregnancy.

PGL, as an extra-adrenal PCC, has a long disease course with slow growth. It prefers to occur in the head and neck, retroperitoneum, and mediastinum. PGL is categorized as functional or nonfunctional based on its clinical manifestations and CA levels in the blood. Fishbein et al^[1]^ reported that PGLs in the neck and skull base were likely derived from parasympathetic ganglia, most of which were nonfunctional, for they didn’t secrete CA. PGL in the chest, abdomen and pelvis commonly stemmed from paraspinal sympathetic nerve chain and were functional because of its ability to secret CA. Functional PGL is mainly characterized by persistent or paroxysmal hypertension, headache, palpitations, and other symptoms such as chest pain, dyspnea, abdominal pain, orthostatic hypotension, pulmonary edema, epilepsy, and even sudden death.^[2]^ Current studies suggest that about 30% of PCC and PGL are associated with germline mutations in the causative gene.

PGL is primarily manifested as paroxysmal hypertension (40–50%), with the “triad” (40–48%) of headache (59–71%), palpitations (50–65%), and hyperhidrosis (50–65%). Therefore, special attention should be paid to hypertension accompanied by the “triad” in clinical diagnosis. The patient reported here presented headache and palpitations when lying or changing her position on the bed in the third trimester, which was probably caused by compression on the tumor from the enlarged uterus while changing her position. Because a variety of tissues and cells in the body were distributed with different subtypes of α and β-adrenergic receptors,^[3]^ hence in addition to hypertension with the “triad,” this pregnant woman also developed abnormal liver function, blood glucose metabolism disorders, and high blood glucose, the presence of which was even earlier than high blood pressure.^[3]^

The first option of pathological diagnosis for PPGL should be the test of plasma free metanephrine (MN) and normetanephrine (NMN)^[3]^ or in the urine, including MN and NMN, the sensitivity and negative result of which have the best prediction value. The sensitivity of plasma free MN could be as high as from 95% to 100%. The test of urinary fractionated MNs has similar sensitivity. Generally, the specialty of PPGL diagnosis could be improved if either MN or NMN in MNs test has increased over 3 folds or both have elevated. Other biochemical tests such as tests of CA, VMA or chromogranin A are not optimal choices for clinical diagnosis of PPGL because of their low accuracy, instead they could be used as auxiliary tools. Moreover, the test results could be impacted by patient’s position, age or medications. For instance, the upper reference limit of NMN level of a patient in sitting position is twice higher than that of a patients in supine position. Stress and caffeine also can result in false positive results. PPGL could be diagnosed by biochemical tests, and localized with imaging technologies. Although pregnancy does not have impacts on the results of biochemical tests, enlarged uterus makes MRI and/or ultrasonic scans be the first choices for anatomical localization of tumors.^[4]^

The clinical manifestations and biochemical test results of the patient in the third trimester reported here was suggestive of PCC. However, MRI scan failed to find or localize the tumor. After consultation to multidisciplinary specialists, she was confirmed with PCC. While undergoing cesarean section, her blood pressure, heart rate and electrocardiogram were all monitored to maintain stable vital signs. Joint efforts of multidisciplinary specialists ensured the safety of the cesarean section process. After surgery, the monitoring of ambulatory blood pressure, body temperature, subjective symptoms as well as the CT scan results of the neck, chest and abdomen revealed tumor lesions in the middle abdomen, suggesting PGL.

Under this condition, genetic testing should be carried out if possible. A total of 20 sensitive causative genes have been reported from 1990 until today,^[3,5]^ whose mutations are associated with 50% of PPGL occurrence, of which 35% to 40% are germline mutations and manifested as hereditary, for example case 3. The pregnant woman in our case is relatively young, resulting in increased probability of pathogenic germline mutations (30–40%). Therefore, she was at greater risk of having multifocal, recurrent and metastatic (malignant) illness, as in cases 10 and 13.

Phenoxybenzamine, a long-acting α-blocker, is the drug of choice for controlling hypertension in PPGL patients. Oral administration of 1 to 5 mg or intravenous pumping (1 mg/min) of phenoxybenzamine is normally safe for fetus. However, it would pass through the placenta, leading to perinatal depression and transient hypotension in infants

After being pathologically confirmed with PGL at the Department of Internal Medicine of our hospital, the pregnant woman right away received β-adrenergic receptor blocker (Labelol), cal^2+^ channel blocker (Adalat), and endogenous calcium antagonist (magnesium sulfate) for 6 days, all of which are commonly used therapeutic medication in the expert consensus with the capacity of effectively reducing adrenergic excitability, slowing heart rate, and lowering blood pressure. She was performed with cesarean section in the lower uterine part due to the presence of “secondary hypertension (PCC?), placental dysfunction, liver dysfunction, and intrahepatic cholestasis.” Preoperative MRI scan failed to localize the tumor, leading to the inability to resect the tumor during the process of cesarean section. Once without the covering of enlarged uterus or fetus cesarean section after cesarean section, immediate CT scanning had specified the tumor site to allow the carry-out of robotic surgery after the completion of α-adrenergic receptor blocker therapy in our Department of Urology. Her prognosis was ideal. As shown in Table 1, 6 pregnant patients did not have long-acting α-blocker treatment for 2 weeks before delivery, specially case 21, who used neither α-blockers nor β-blockers. Their ovarian teratomas and PGL were cleared at the same time during full-term cesarean section. Therefore, the combined use of β-adrenergic receptor blockers and cal^2+^ channel blockers plus Magnesium Sulfate could inhibit CA from releasing. It is a safe and effective therapy to maintain perioperative hemodynamics in patients, and proved to be ideal in the treatment of PGL in patients during pregnancy, as supported by their satisfying prognosis. It is also a new thought for before-delivery PGL treatment.

As stated in the literature, if patients whose tumors were not diagnosed or unresected before delivery, the mortality of her undergoing vaginal delivery was higher than experiencing cesarean section (31% vs 19%). Hence, cesarean section under close monitoring of an experienced anesthesiologist is a relatively good way to terminate pregnancy of patients whose tumors are not removed before delivery.^[6]^ Even as patients have received medication before delivery, abdominal pressure and contractions during labor would still increase the risks of failures in delivery in pregnant women with PGL. So, it is wise to choose a proper delivery mode for pregnant women with PGL according to their tumor sites, clinical symptoms and obstetric conditions. We found 3 patients who had successfully experienced vaginal delivery after tumor removal before or during pregnancy.^[7–9]^ Ten patients underwent cesarean section and had their tumors removed after surgery.

Laparotomy, laparoscopy, and robotic surgery can be applied for the treatment of tumors according to their locations.^[10]^ The hypertension in most PPGL patients could be cured and their CA levels restored normally in one week after successful tumor removal. As many as 75% of patients had normal blood pressure in one month after surgery, and the remaining 25% still had high blood pressure, but lower than before surgery. Satisfactory outcome was achieved in them with the use of usual antihypertensive drugs The survival rate of patients with nonmetastatic PPGL was above 95% and the recurrence rate below 10%. And the 5-year survival rate of patients with metastatic PPGL was lower than 50%. Life-time follow-ups seems to be necessary for PPGL patients. Therefore, it is recommended that they have at least one recheck each year. For patients who have genetic mutations and metastatic PPGL, follow-up is recommended every 3 to 6 months. Genetic testing and regular physical examination are also suggested for their immediate families.^[3]^ The measurements for follow-ups should include symptoms, vital signs, plasma/urinary MNs, and imaging check when necessary. The patient reported here was observed with stable blood pressure, heart rate and blood glucose after surgery, without oral administration of antihypertensive medications. Her postoperative blood check showed normal MNs, and genetic testing indicated low risks of recurrence.

Limited by a small case number and unknown pathophysiological mechanisms of pregnancy complications, it is really hard to localize PGL in patients during pregnancy, particularly during their third trimester. Six out of the 21 patients were misdiagnosed with hypertension or diabetes. The tumor of the patient reported here was failed to be localized before delivery, plus the other 6 whose tumors were localized only after labor. Also, we are unable to illustrate the relation between the gestational week on diagnosis and pregnancy outcomes due to the small case number. However, patients whose symptoms were under control or whose tumors were surgically cleared during pregnancy produced satisfactory prognosis. So, it is crucial for PGL patients to have early diagnosis and treatment. Because PGL during pregnancy is extremely rare, so a larger sample size is needed to guide in the making of evidence-based optimal treatment plan and determining the long-term outcome for such patients.

In general, pregnant women with PGL identified in the third trimester could still obtain good maternal and fetal outcomes if physicians have more information about the disease, PGL is diagnosed early and accurately, and the pregnancy is terminated at a proper time according to the obstetric conditions of patients. It is worth noting that preparations of combined use of α- and β-blockers 2 weeks before cesarean section is unnecessary to be overstressed if patients were not to remove their tumors during the section.

## Author contributions

**Conceptualization:** Mei Xiao.

**Formal analysis:** Xiaohong Chen.

**Funding acquisition:** Miaomiao Chen.

**Investigation:** Ling Yu.

**Methodology:** Wen Zhong Yang, Chengcheng Jiang.

**Project administration:** Shu Guo Du.

**Supervision:** Quan Gan.

**Writing – original draft:** Lei Zhao.

## Supplementary Material



## References

[1] Fishbein L Orlowski R Cohen D. Pheochromocytoma/paraganglioma: review of perioperative management of blood pressure and update on genetic mutations associated with pheochromocytoma. J Clin Hypertens (Greenwich). 2013;15:428–34. 23730992 10.1111/jch.12084 PMC4581847

[2] Koroscil TM McDonald S Stutes S. Use of fluorine-18-labelled deoxyglucose positron emission tomography with computed tomography to localize a paraganglioma in pregnancy. South Med J. 2010;103:1238–42. 21037527 10.1097/SMJ.0b013e3181eda0de

[3] Wang T Xie L. Interpretation of Expert Consensus on the diagnosis and treatment of pheochromocytoma and paraganglioma (2020 Edition). Chin J Hypertension 2021;29:708–14.

[4] Aygun N Uludag M. Pheochromocytoma and paraganglioma: from clinical findings to diagnosis. Sisli Etfal Hastan Tip Bul 2020;54:271–80. 33312023 10.14744/SEMB.2020.14826 PMC7729715

[5] Taieb D Yang C Delenne B. First report of bilateral pheochromocytoma in the clinical spectrum of HIF2A-related polycythemia-paraganglioma syndrome. J Clin Endocrinol Metab. 2013;98:E908–13. 23539726 10.1210/jc.2013-1217 PMC3644612

[6] Wiseman D Lakis ME Nilubol N. Precision surgery for pheochromocytomas and paragangliomas. Horm Metab Res. 2019;51:470–82. 31307109 10.1055/a-0926-3618 PMC8572371

[7] Chu PY Burks ML Solorzano CC. Robotic paraganglioma resection in a pregnant patient. AACE Clin Case Rep. 2020;6:e197–200. 32984520 10.4158/ACCR-2019-0558 PMC7511102

[8] Radfar MH Shakiba B Afyouni A. Laparoscopic management of paraganglioma in a pregnant woman: a case report. Int Braz J Urol. 2018;44:1032–5. 29570256 10.1590/S1677-5538.IBJU.2017.0698 PMC6237544

[9] Mabrouk MY Jabi R Bouzayan L. Management of a left lateral aortic paraganglioma during pregnancy: a rare case report. Cureus 2021;13:e19221. 34873546 10.7759/cureus.19221 PMC8640192

[10] Podolsky ER Feo L Brooks AD. Robotic resection of pheochromocytoma in the second trimester of pregnancy. JSLS 2010;14:303–8. 20949656 10.4293/108680810X12785289145006 PMC3043591

[11] Pambinezhthu F Hamza N Al Kharusi M. Hereditary paraganglioma in an Omani family. Oman Med J. 2021;36:e229. 33628464 10.5001/omj.2021.10 PMC7897353

[12] Luk A Ma RC Lam CW. A 21-year-old pregnant woman with hypertension and proteinuria. PLoS Med. 2009;6:e1000037. 19243216 10.1371/journal.pmed.1000037 PMC2646781

[13] Han Q Lv G. A case of recurrence of ectopic pheochromocytoma in pregnancy. Tianjin Med J. 1996;24:186.

[14] Tomasian A Lai C Ruehm S. Cardiovascular magnetic resonance and PET-CT of left atrial paraganglioma. J Cardiovasc Magn Reson. 2010;12:1. 20047692 10.1186/1532-429X-12-1 PMC2817869

[15] Kim HJ Yang SH Yang SH. Extra-adrenal paraganglioma masquerading as severe preeclampsia. Obstet Gynecol Sci. 2018;61:520–3. 30018907 10.5468/ogs.2018.61.4.520 PMC6046359

[16] Dusitkasem S Herndon BH Paluzzi D. From bad to worse: paraganglioma diagnosis during induction of labor for coexisting preeclampsia. Case Rep Anesthesiol. 2017;2017:5495808. 28197344 10.1155/2017/5495808 PMC5286480

[17] Yang C Ma H Qiang W. A case of twin pregnancy in a patient with non-adrenal pheochromocytoma. Chin J Surg. 2004:58.14989853

[18] Liu T Zhang H Yang R. Ectopic pheochromocytoma misdiagnosed as pregnancy-induced hypertension syndrome in 1 case. Chin J Hypertension 2015;23:95–7.

[19] Mohamed RS Antonypillai CN Mahendran H. Paraganglioma presenting as hypertension during pregnancy, proteinuria, thrombocytosis, and diabetes mellitus: a case report. J Med Case Rep. 2021;15:352. 34238353 10.1186/s13256-021-02923-1 PMC8268307

[20] Narechania S Bath A Ghassemi L. Paraganglioma presenting as postpartum fever of unknown origin. Case Rep Endocrinol. 2015;2015:864719. 26236513 10.1155/2015/864719 PMC4508462

[21] Makis W McCann K McEwan AJ. The challenges of treating paraganglioma patients with (177)Lu-DOTATATE PRRT: catecholamine crises, tumor lysis syndrome and the need for modification of treatment protocols. Nucl Med Mol Imag 2015;49:223–30. 10.1007/s13139-015-0332-6 PMC4532685 26279696

[22] Fraser LA Kiaii B Shaban J. Cardiac pheochromocytoma presenting during pregnancy. BMJ Case Rep 2010;2010:bcr0420102890. 10.1136/bcr.04.2010.2890 PMC3028230 22791480

[23] Zhang H Yan J Jiang L. A case report of paraganglioma in the third trimester of pregnancy and literature review. Progr Mod Obstetr Gynecol 2017;26:157–9.

