# Supplementary material for: Ectopic pheochromocytomas in the third trimester: A case report and literature review
Source: Medicine (Baltimore). 2023 Feb 2;103(5):e36127. doi: 10.1097/MD.0000000000036127 (PMC10843473; doi:10.1097/MD.0000000000036127)
Supplement: Supplementary file 1 [file medi-103-e36127-s001.docx]

**Table S1. Antihypertensive medications of pregnant patients who didn’t undergo EPCC removal before delivery**

| Case No. | Publication time | Region | Diagnosis time | Gestational weeks | Pregnancy termination signs | Mode of delivery | Timing of tumor resection | Tumor size | Site | Note |
| --- | --- | --- | --- | --- | --- | --- | --- | --- | --- | --- |
| 1 (our case) |  | Wuhan, China | At 34^+1^ weeks | 35^+1^ weeks | Placental insufficiency, ICP | Cesarean section | Robotic surgery 6 weeks after delivery | 6.0×4.5×6.6 | On the superior right of the abdominal aorta in the middle abdomen |  |
| 2^[3]^ | 2020 | Denver, USA | During IVF-ET | Unknown | - | Unknown | Before conception | 0.42 | In the center of left ovarian teratoma | IVF oocyte retrieval |
| 3^[4]^ | 2021 | Oman | At 4 weeks | 37 weeks | Hypertension | Cesarean section | 3 months after delivery | 10.5×5.3×3.3 | Along the para-aortic bifurcation down to the pelvis and the anterior | Family history of PPGL |
| 4^[5]^ | 2020 | Tennessee, USA | At 12 weeks | 39^+1^ weeks | Spontaneous labor | Spontaneous delivery | Robotic surgery at 8 weeks of gestation | 4.8 ×4.3 | In the left retroperitoneum near aorta, just below adrenal gland |  |
| 5^[6]^ | 2015 | Mumbai, India | At 9 weeks | Full term | Extended latent period | Cesarean section | Partial bladder resection in the second trimester | 6.5×5.5 | From the anterior vaginal cavity to a little bit further through the inferior bladder wall |  |
| 6^[7]^ | 2005 | Ya'an, Sichuan province, China | At 3 months |  | - | - | - | 9.8×11.3×13.9 | On the left superior abdomen, closely related to the bottom of the pancreas, by the side of the retroperitoneal abdominal aorta |  |
| 7^[8]^ | 2018 | Tehran, Iran | At 17 weeks | 39 weeks | Spontaneous delivery | Spontaneous delivery | Laparoscopic surgery at 19 weeks | 3×3.5 | In the left renal hilum |  |
| 8^[9]^ | 2021 | Morocco | At 20 weeks |  | - | — | Laparoscopic surgery at 25 weeks | 3.6×3.3 | By the side of the lateral left aorta of the retroperitoneum |  |
| 9^[10]^ | 2009 | Hongkong, China | 24 weeks | 38 weeks | Previous cesarean section | Cesarean section | Abdominal resection 4 months postpartum | 4.2 | In the mid-abdominal aorta | Family history of hypertension |
| 10^[11]^ | 1996 | Tianjing, China | At 25 weeks | 30 weeks | Mild water accumulation in both kidneys with mtabolic ketoacidosis | Cesarean section | Recurred PGL during cesarean section | 2.5×1×0.5;3×2×1；1.5×1×1;0.5×1.5×1 | Four tumors, one in the anterior left sacral promontory and one in the superior left common iliac artery bifurcation, two in the left abdominal aortas. | Pheochromocytoma removal history in 1984 |
| 11^[12]^ | 2021 | Morocco | At 31 weeks | 37 weeks | Spontaneous labor | Spontaneous delivery | Laparoscopic surgery at 32 weeks | 3.6×3.3 | In the left lateral abdominal aorta |  |
| 12^[13]^ | 2010 | LA, USA | In the third trimester | - | Hypertensive crisis | Cesarean section | 6 weeks after delivery | 4.5×2.7×3.9 | In the posterior mediastinal area near left atrium |  |
| 13^[14]^ | 2018 | Seoul, South Korea | At 37 weeks | 37 weeks | Prolonged monitoring of decelerated fetal heart rate | Cesarean section | Post-delivery laparotomy | 4 | In the para-aortic area near left renal hilum | Severe preeclampsia |
| 14^[15]^ | 2017 | Ohio, USA | At 33^+5^ weeks | 33^+5^ weeks | Pathologically diagnosed with PGL at induction of labor on day 7 of hospitalization | Changing to cesarean section when the cervix dilated 5cm. | Surgery after 2 weeks of delivery | 4.7×6.4 | Next to the left aorta | No triad on admission |
| 15^[16]^ | 2004 | Tianjing, China | At 34 weeks | 36 weeks | Twins  Pheochromocytoma | Cesarean section | Mass removal while having cesarean section | 6×5.0×4.5 | At the very bottom of left kidney, next to the left abdominal aorta. | Twins |
| 16^[17]^ | 2015 | Shijiazhuang, China | At 2 months after delivery | 40 weeks | Hypertension during pregnancy | Cesarean section | Mass removal after 2 months of gestation | 4×4×4 | In the retroperitoneal space below bifurcation of abdominal aorta | Hospitalization due to severe preeclampsia |
| 17^[18]^ | 2021 | Kangti, Sri Lanka | Postpartum | 33 weeks | Hypertensive crisis | Cesarean section | Surgery after delivery | 5.1×5.2×4.7 | In the posterior uterus | Hospitalization due to preeclampsia, diabetes and thrombocytosis |
| 18^[19]^ | 2015 | Cleveland, USA | 2 days after delivery | 37 weeks | The presence of decelerated fetal heart rate at the hypertension-caused induction | Cesarean section | Surgery after delivery | 9.2×14×12.5 | In the right retroperitoneum | Spreading to the lung and spines |
| 19^[20]^ | 2015 | Alberta, Canada | Postpartum | 28 weeks | Preeclampsia | Cesarean section | Unresected, hereditary SDH-C mutation c.397C > T | 7.4×5.6 | In the pulmonary arteries, aorta, SVC and atria | Targeted radioisotope therapy |
| 20^[21]^ | 2010 | Western Ontario, Canada | Postpartum | 34 weeks | Malignant hypertension | Cesarean section | Surgery after delivery | - | In the right atrioventricular of coronary arteries |  |
| 21^[22]^ | 2017 | Fujian, China | Postpartum | 38^+6^ weeks | Fetal distress, ICP | Cesarean section | Hypertension-induced ovarian mass removal during cesarean section | 5×5×4 | In the posterior of left ovary | No hypertension during pregnancy |
